# Whole-Body and Hepatic Insulin Resistance in Obese Children

**DOI:** 10.1371/journal.pone.0113576

**Published:** 2014-11-20

**Authors:** Lorena del Rocío Ibarra-Reynoso, Liudmila Pisarchyk, Elva Leticia Pérez-Luque, Ma. Eugenia Garay-Sevilla, Juan Manuel Malacara

**Affiliations:** Department of Medical Sciences, University of Guanajuato, Campus León, 20 de Enero 929, León Guanajuato, México; Dasman Diabetes Institute, Kuwait

## Abstract

**Background:**

Insulin resistance may be assessed as whole body or hepatic.

**Objective:**

To study factors associated with both types of insulin resistance.

**Methods:**

Cross-sectional study of 182 obese children. Somatometric measurements were registered, and the following three adiposity indexes were compared: BMI, waist-to-height ratio and visceral adiposity. Whole-body insulin resistance was evaluated using HOMA-IR, with 2.5 as the cut-off point. Hepatic insulin resistance was considered for IGFBP-1 level quartiles 1 to 3 (<6.67 ng/ml). We determined metabolite and hormone levels and performed a liver ultrasound.

**Results:**

The majority, 73.1%, of obese children had whole-body insulin resistance and hepatic insulin resistance, while 7% did not have either type. HOMA-IR was negatively associated with IGFBP-1 and positively associated with BMI, triglycerides, leptin and mother's BMI. Girls had increased HOMA-IR. IGFBP-1 was negatively associated with waist-to-height ratio, age, leptin, HOMA-IR and IGF-I. We did not find HOMA-IR or IGFBP-1 associated with fatty liver.

**Conclusion:**

In school-aged children, BMI is the best metric to predict whole-body insulin resistance, and waist-to-height ratio is the best predictor of hepatic insulin resistance, indicating that central obesity is important for hepatic insulin resistance. The reciprocal negative association of IGFBP-1 and HOMA-IR may represent a strong interaction of the physiological processes of both whole-body and hepatic insulin resistance.

## Introduction

Insulin resistance (IR) is an important metabolic alteration that is frequently associated with obesity and appears to be the primary mediator of metabolic syndrome [1]. IR and persistent hyperinsulinemia are found in a variety of other medical conditions, such as dyslipidemia and hypertension, mainly in obese children as early as 3 to 5 years of age [2].

IR is mediated by genetic and acquired pathophysiological factors. At early stages, IR appears to affect various molecular pathways, predominantly inflammation, at the cellular level in muscle, adipocytes, and endothelial cells [3]. Counter-regulatory hormone alteration is another factor involved in IR; in rodents, glucagon suppresses hepatic glucose production through activity regulated at the mediobasal hypothalamus through the vagus nerve [4].

IR has been considered to be either whole body or central (hepatic). The periphery IR consists of impaired glucose uptake and consumption mainly in muscle and fat and is measured by Homeostatic Model Assessment-IR (HOMA-IR) [5]. Hepatic IR results in unrestrained liver glucose production [6]. The following heterogeneous signaling pathways participate in this process: liver cytohesin is required for insulin signaling and its inhibition by SecinH3 [7]; activation of NOTCH receptors results in lipolysis [8] and hepatic glucose production [9]; the target of rapamycin complex (TORC2) pathway also modulates glucose expenditure [10]; and sterol regulatory element-binding protein-1 (SREBP-1) mediates insulin's effect on fatty acid synthesis [11]. In a parallel process, saturated and unsaturated fats lead to hepatic accumulation of diacylglycerols, activation of protein kinase Cε (PKCε), and impairment of insulin-stimulated insulin receptor substrate 2 (IRS-2) signaling [12]. An important recently identified factor for hepatic IR is high glucose or fructose intake [13].

Insulin-like growth factor binding protein-1 (IGFBP-1) is secreted in the liver under insulin regulation and has been proposed as a specific marker of hepatic IR [14], [15] and as a convenient and sensitive marker for hepatic IR in children [16].

Obesity is frequently associated with the development of non-alcoholic fatty liver disease (NAFLD) [17] and is a major factor in the pathogenesis of type 2 diabetes [18]. However, the association of NAFLD with hepatic or whole-body IR is not well defined.

In this work, we studied the factors associated with whole-body and hepatic IR as assessed by IGFBP-1 blood levels in obese children and the potential relationship with ultrasonographic assessment of NAFLD. For better estimation of the association between children's obesity and IR, we compared obesity metrics such as body mass index (BMI), visceral adiposity index (VAI), and waist-to-height ratio.

## Materials and Methods

Between August 2011 and April 2012, we recruited 182 obese children, six to eleven years old, from grammar schools in the city of León in central Mexico, which is representative of the population of our region. The study group included children with BMI higher than the equivalent of 30 kg/m^2^ for an adult after adjusting for gender and age according to the International Tables reported by Cole et al. [19]. The selected children did not have clinical evidence of hypothyroidism, chronic infections, or congenital or metabolic diseases.

### Ethics Statement

The nature and purpose of the study was explained to the children and their parents. If both the children and parents or tutor accepted, the parents signed an informed consent form; confidentiality of individual results was guaranteed. The study was approved by the Ethics Committee of the Department of Medical Research. University of Guanajuato (CEDCM-2009-9).

### Data collection

Data were collected by direct questioning of the children and at least one of their parents. We collected the family history of obesity and diabetes as well as the mother's BMI. Children's sleep duration and exercise levels were also recorded. Acanthosis nigricans was registered as a score from 0 to 4.

Weight and standing height were obtained with indoor clothing and without shoes using a roman-type scale and a Harpenden stadiometer in order to calculate the BMI. Waist girth was measured with indoor clothes using a non-extendible flexible tape at the midpoint of the last rib and the iliac crest. Waist-to-height ratio and VAI were calculated. The VAI formula was as follows: for boys  =  (waist girth/39.68+(1.88×BMI)) ×(triglycerides/1.03) × (1.31/HDL-cholesterol) and for girls  =  (waist girth/36.58+(1.89×BMI)) ×(triglycerides/0.81) × (1.52/HDL-cholesterol) [20]. Skin fold thickness was obtained at bicipital, tricipital, suprailiac and subscapular sites to calculate body density as follows: for boys  = 1.1533-(0.0643× log ∑ (4 measurements of skin fold thickness)) and for girls  = 1.1369-(0.0598× log ∑ (4 measurements)) [21], [22]. The percent body fat [23] was calculated as {4.95/body density - 4.5} ×100.

A venous blood sample was obtained after twelve hours of fasting to measure hormone and metabolite levels. Glucose, triglycerides, and cholesterol were measured by conventional methods. Insulin, leptin and adiponectin were measured by radioimmunoassay using a Millipore kit (St. Charles, Mo) with intra- and interassay variation coefficients of 4.4% and 6.0% for insulin, 3.4% and 3.6% for leptin, and 6.2% and 9.2% for adiponectin. Insulin-like growth factor I (IGF-I) and IGFBP-1 were measured by ELISA (Mediagnost, Reutlingen, Germany) with intra- and interassay variation coefficients of 5.1% and 6.8% for IGF-I and 6.2% and 7.4% for IGFBP-1.

### Liver ultrasound

The presence of a fatty liver was assessed by ultrasound, which is considered an appropriate and practical method [24]. The procedure was carried out by an experienced physician radiologist using a General Electric Logic 400 MD Doppler Color with a convex transducer of 3.6 MHz. The results were assessed by two experienced radiologist and classified into the following four groups by the extent of liver steatosis: negative, slight, moderate and severe, according to Mittelstaedt [25].

### Statistical Analysis

Data are shown as the means and standard deviations (SD). Whole-body IR was evaluated with HOMA-IR, taking 2.5 as the cut-off point as proposed for prepubertal children [26]. Considering that a low level of IGFBP-1 has been proposed to be a marker of hepatic IR [14], we took the 3^rd^ quartile of IGFBP-1 as the cut-off point for hepatic IR. Groups with and without IR were compared by means of a two-tailed Student's t-test for independent samples or the Mann-Whitney U test when non-parametric data were obtained. The Chi-square test was used to analyze fatty liver and acanthosis nigricans scores.

We compared the groups of insulin resistance and **NAFLD groups** by ANOVA.

Factors associated with HOMA-IR and IGFBP-1 were analyzed by a generalized linear model with stepwise elimination of non-significant variables. Using the HOMA-IR results as the dependent variable, we tested the following candidate regressors: individual estimators of obesity (BMI, waist-to-height ratio, and VAI, successively), age, sex, fatty liver score, leptin, IGF-I, IGFBP-1, total cholesterol, triglycerides, adiponectin, sleep duration and mother's BMI. Using IGFBP-1 as the dependent variable, we tested the following candidate regressors: estimators of obesity, age, sex, leptin, adiponectin, HOMA, IGF-I, total cholesterol, triglycerides, mother's BMI, fatty liver score and hours of sleep. Statistica 7.0 for Windows (Statsoft, Tucson AZ) was used for the analyses. P<0.05 was considered significant.

## Results

The 182 obese children had a mean age of 9.2±1.4 years, BMI 27.2±3.6, waist girth 89.0±11.5 cm, waist-to-height ratio 0.6±0.1, VAI 1.2±0.7, and percent body fat 38.3±2.8%. In regard to pubertal activation, only 7 girls from 10- to 11-year-old exhibited pubertal activation. HOMA-IR was 5.3±2.3, leptin 27.4±12.7 ng/ml, glucose 4.74±0.59 mmol/L, insulin 25.2±10.0 µIU/ml, IGF-I 26.9±14.2 nmol/L, and adiponectin 15.5±8.1 ng/ml. Overall, 18.4% of the children had at least one parent with a diagnosis of Type 2 Diabetes Mellitus (Type2 DM). A total of 167 (91.8%) obese children had HOMA-IR values higher than 2.5 and were therefore considered to have whole-body IR. IGFBP-1 was measured in 171 children with a mean value of 5.1±4.4 ng/ml. Among these children, 128 were considered to have hepatic IR (74.8%).

### Comparison of groups with and without whole-body IR

As shown in table 1, subjects without whole-body IR (8.2%) were younger and had lower BMI, waist-to-height ratio, VAI, percent total body fat, triglycerides and leptin levels but increased IGFBP-1 levels. When comparing children with high HOMA-IR vs low HOMA-IR were found marginal differences for acanthosis nigricans, but unexpectedly there was no difference for fatty liver.

**Table 1:** Characteristics of the obese children with or without whole body IR (HOMA-IR cut off point 2.5).

	High HOMA-IR	Low HOMA-IR	t value	P value
	Mean±SD	Mean±SD		
	N = 167	N = 15		
Age, years	9.26±1.34	8.10±1.09	3.23	<0.002
BMI, kg/m^2^	27.44±3.58	24.28±2.62	3.33	<0.001
waist-to-height ratio	0.64±0.07	0.60±0.04	2.18	<0.03
VAI	1.30±0.75	0.69±0.31	3.16	<0.002
Percent body fat	38.61±2.70	35.17±2.20	4.80	<0.000003
Triglycerides, mmol/L	1.52±0.63	1.08±0.42	2.66	<0.009
Total cholesterol, mmol/L	3.95±0.74	3.96±0.77	−0.07	0.95
HDL cholesterol, mmol/L	1.06±0.24	1.15±0.22	−1.34	0.18
Leptin, ng/ml	28.19±12.80	18.00±6.04	3.05	<0.003
IGF-I, nmol/L	22.23±14.74	23.37±5.27	1.01	0.31
IGFBP-1, ng/ml	4.62±3.99	10.08±5.17	−4.93	<0.000002
Adiponectin, ng/ml	15.18±7.91	18.51±8.53	−1.50	0.13
Sleep, hours/day	9.16±1.01	9.47±1.14	−1.10	0.27
Exercise, min/week	222.88±225.53	263.33±217.18	−0.67	0.51
Mother's BMI	32.41±6.36	29.58±5.29	1.55	0.12
	**N (%)**	**N (%)**	**Chi-square**	**p value**
Without fatty liver	77 (57.46%)	8 (57.14%)	1.1	0.78
Slight	36 (26.87%)	5 (35.71%)		
Moderate	17 (12.69%)	1 (7.14%)		
Severe	4 (2.99%)	0 (0%)		
Without acantosis nigricans	45 (31.03%)	9 (75%)	9.74	<0.04
Grade 1	55 (37.93%)	2 (16.67%)		
Grade 2	28 (19.31%)	1 (8.33%)		
Grade 3	4(2.76%)	0 (0%)		
Grade 4	13(8.97)	0 (0%)		

Characteristics of obese children with or without whole-body IR (HOMA-IR cut off point 2.5). Body mass index (BMI), visceral adiposity index (VAI), insulin-like growth factor binding protein-1 (IGFBP-1), insulin-like growth factor I (IGF-I).

### Comparison of groups with low and high IGFBP-1 values

The circulating levels of IGFBP-1 had a median of 4.33 ng/ml, lower quartile of 2.0 ng/ml and upper quartile of 6.67 ng/ml. We considered subjects with an IGFBP-1 value lower than 6.67 ng/ml as having hepatic IR. The comparison of children with low and high IGFBP-1 levels is shown in table 2. Similar to the group with high HOMA-IR, children with hepatic IR were older and had higher BMI, VAI, percent body fat and leptin. In contrast to the different triglyceride levels seen in the high vs low HOMA-IR groups, triglyceride levels were similar in children with and without hepatic IR. Children with hepatic IR had higher leptin, IGF-I and insulin levels and lower adiponectin and reduced sleep duration. Fatty liver and acanthosis nigricans were not associated with IGFBP-1 levels.

**Table 2:** Characteristics of the obese children with or without hepatic IR (IGFBP-1 cut off point 6.67 ng/ml).

	With Hepatic IR	Without Hepatic IR	t value	P value
	(three lowest quartiles)	(highest quartile)		
	Mean±SD	Mean±SD		
	N = 128	N = 43		
Age, years	9.34±1.26	8.37±1.35	4.24	<0.00004
BMI, kg/m^2^	27.51±3.41	25.48±3.15	3.40	<0.0008
Waist-to-height ratio	0.64±0.07	0.63±0.05	0.52	0.60
VAI	1.30±0.71	0.94±0.49	3.00	<0.003
Percent body fat	38.71±2.61	36.89±2.88	3.86	<0.0002
Glucose, mmol/L	4.75±0.60	4.65±0.53	0.95	0.34
Triglycerides, mmol/L	1.51±0.62	1.34±0.55	1.61	0.11
Total cholesterol, mmol/L	3.97±0.72	3.94±0.75	0.25	0.80
HDL cholesterol, mmol/L	1.07±0.24	1.08±0.25	−0.32	0.75
Leptin, ng/ml	29.24±13.22	21.28±8.63	3.69	<0.0003
Insulin, µIU/ml	27.27±9.21	17.74±8.09	6.05	<0.0000001
IGF-I, nmol/L	27.73±13.01	21.09±10.87	3.01	<0.003
Adiponectin, ng/ml	14.82±7.78	17.98±7.55	−2.31	<0.02
Sleep, hours/day	9.02±0.91	9.72±1.17	−4.06	<0.00007
Exercise, min/week	216.75±221.24	258.60±240.44	−1.05	0.3
Mother's BMI	32.39±6.63	31.29±5.65	0.83	0.41
	**N (%)**	**N (%)**	**Chi-square**	**P value**
Without fatty liver	57 (55.34%)	23 (62.16%)	1.11	0.77
Slight	28 (27.18%)	10 (27.03%)		
Moderate	15 (14.56%)	3 (8.11%)		
Severe	3 (2.91%)	1 (2.7%)		
Without acantosis nigricans	34 (30.36%)	19 (52.78%)	8.31	0.08
Grade 1	42 (37.50%)	8 (22.22%)		
Grade 2	24 (21.43%)	5 (13.89%)		
Grade 3	8 (7.14%)	4 (11.11%)		
Grade 4	4 (3.57%)	0 (0%)		

Characteristics of obese children with or without hepatic IR (IGFBP-1 cut off point 6.67 ng/ml). body mass index (BMI), visceral adiposity index (VAI), insulin-like growth factor binding protein-1 (IGFBP-1), insulin-like growth factor I (IGF-I).

### Factors associated with HOMA-IR

The generalized linear model tested three indices of adiposity and showed positive associations with mother's BMI, children's BMI, triglycerides and leptin levels and a negative association with IGFBP-1 levels. After testing for gender as a confounding variable, male gender was negatively associated with HOMA-IR levels in the total group (Table 3).

**Table 3:** Factors associated with HOMA-IR.

		Estimate±SD	Wald	P value
Testing the inclusion of waist-to-height ratio				
Triglycerides, mmol/L		0.002±0.0005	10.38	<0.001
Leptin, ng/ml		0.006±0.003	5.97	<0.01
IGFBP-1, ng/ml		−0.06±0.01	32.6	<0.0000001
Mother's BMI		0.01±0.004	12.08	<0.0005
Testing the inclusion of BMI				
BMI, kg/m^2^		0.02±0.008	7.96	<0.005
Triglycerides, mmol/L		0.001±0.0005	6.81	<0.009
IGFBP-1, ng/ml		−0.05±0.01	22.87	<0.000002
Mother's BMI		0.01±0.005	9.97	<0.002
Sex	boys	−0.07±0.03	4.59	<0.03
Testing the inclusion of VAI				
Triglycerides, mmol/L		0.002±0.0005	10.38	<0.001
Leptin, ng/ml		0.006±0.003	5.97	<0.01
IGFBP-1, ng/ml		−0.06±0.01	32.6	<0.0000001
Mother's BMI		0.01±0.004	12.08	<0.0005

Factors associated with HOMA-IR were analyzed by means of the generalized linear model, testing three different types of adiposity index. Body mass index (BMI), visceral adiposity index (VAI), insulin-like growth factor binding protein-1 (IGFBP-1).

### Factors associated with IGFBP-1 serum levels

The generalized linear model tested three indices of adiposity and showed negative associations with waist-to-height ratio, age, leptin, HOMA-IR and IGF-I levels (Table 4).

**Table 4:** Factors associated with IGFBP-1.

	Estimate±SD	Wald	p
Testing the inclusion of waist-to-height ratio			
Waist-to-height ratio	−2.15±0.88	5.92	<0.01
Age, years	−0.13±0.03	15.38	<0.00009
Leptin, ng/ml	−0.02±0.005	14.72	<0.0001
HOMA-IR	−0.15±0.025	34.82	<0.0000001
IGF-I, nmol/L	−0.004±0.0009	24.64	<0.000001
Testing the inclusion of BMI			
Age, years	−0.15±0.03	20.01	<0.0000001
Leptin, ng/ml	−0.02±0.005	17.26	<0.00003
HOMA-IR	−0.15±0.03	36.10	<0.0000001
IGF-I, nmol/L	−0.004±0.0008	20.59	<0.000006
Testing the inclusion of VAI			
Age, years	−0.15±0.03	20.01	<0.0000001
Leptin, ng/ml	−0.02±0.005	17.26	<0.00003
HOMA-IR	−0.15±0.03	36.10	<0.0000001
IGF-I, nmol/L	−0.004±0.0008	20.59	<0.000006

Factors associated with IGFBP-1 analyzed by means of the generalized linear model, testing three different types of adiposity index. Body mass index (BMI), visceral adiposity index (VAI), insulin-like growth factor binding protein-1 (IGFBP-1), insulin-like growth factor I (IGF-I).

The triglyceride level was not independently associated with IGFBP-1; however, after repeating the analysis with HOMA-IR excluded, an association with triglycerides appeared (p<0.004).

### Comparison of groups with high or low HOMA-IR and IGFBP-1

One hundred twenty-five children (73.1%) had high HOMA-IR and low IGFBP-1, interpreted as indicating whole-body insulin resistance and hepatic IR. Low HOMA-IR and high IGFBP-1 was found in 12 children (7.0%), consistent with the absence of both whole-body IR and hepatic IR. High HOMA-IR and high IGFBP-1, indicative of whole-body IR, was found in 31 children (18.1%). Three children (1.8%) had low HOMA-IR and low IGFBP-1, indicating only hepatic IR.

We compared these groups by ANOVA. The group without any form of IR were significantly younger (F = 7.11, p<0.0002) and had lower BMI (F = 5.74, p<0.0001), waist girth (F = 6.84, p<0.00002), VAI (F = 4.96, p<0.002), leptin (F = 5.82, p<0.003), compared with the group with both types of IR. The group without any type IR had lower waist girth (F = 6.84, p<0.04) than the group with only hepatic-IR. Additionally, the group without any type of IR had lower BMI (F = 5.74; p<0.02) and waist girth (F = 6.84, p<0.003), than the group with only whole-body IR. The group with only whole-body IR was younger (F = 7.11, p<0.002) and had lower VAI (F = 4.96, p<0.04), leptin (F = 5.82, p<0.005) and IGF-I levels (F = 3.42, p<0.002) than the group with both types of IR. (Table S1).

### Comparison of NAFLD groups

We carried out a liver ultrasound in a total of 148 children and found that 85 did not have fatty liver, 41 had slight, 18 had moderate and four had severe fatty liver. Comparing the characteristics of these groups, we found that in children without fatty liver, had lower age (F = 3.49, p<0.02), BMI (F = 6.49, p<0.0004), waist girth (F = 2.92, p<0.04) and leptin levels (F = 5.56, p<0.001). We did not find differences among the groups in terms of HOMA-IR, IGFBP-1, waist-to-height ratio, VAI, triglycerides, total cholesterol, HDL cholesterol and adiponectin. (Table S2).

## Discussion

In this work, we compared whole-body and hepatic IR. The evaluation of hepatic IR was based on IGFBP-1 levels. There are currently no criteria for a cut-off point to discriminate between the different extents of low IGFBP-1 levels and diagnose hepatic IR. Intuitively, we proposed that at least three quarters of obese children have hepatic IR, so the upper quartile was considered not to have hepatic IR. These results should support further work to define a more appropriate cut-off point for hepatic IR diagnosis using IGFBP-1 levels.

IR is a metabolic disorder associated with metabolic syndrome [27]. The hormone and metabolic profile of our group of obese children is similar to those described in other reports [28]. Overall, 73.1% had whole-body and hepatic IR and only 7% had normal HOMA-IR and IGFBP-1 values, representing the expected profile for metabolically healthy subjects [29].

In univariate analysis, children without whole-body or hepatic IR were younger, as reported in a previous work [30]; this may indicate that the benign metabolic profile is transient and therefore more prevalent in younger children. Prospective studies are necessary to define the stability of insulin sensitivity.

In an attempt to understand the significance of hepatic IR, we compared factors associated with HOMA-IR and IGFBP-1. A reciprocal negative association of IGFBP-1 and HOMA-IR has also been reported in studies in children [31] and adults [32]. This represents the strong interaction between both physiopathological processes.

Other than the reciprocal association between both types of IR, leptin was the only factor associated with both whole-body IR and hepatic IR. The role of leptin in IR is a controversial subject. In our work, we found leptin was more strongly associated with hepatic IR. German et al. proposed that leptin improves hepatic sensitivity to insulin by means of hypothalamic signaling, an effect blocked by selective hepatic vagotomy [33]. Moreover, central leptin signaling stimulates fatty acid oxidation in white adipose tissue [34] thus controlling lipogenesis [35]. This effect has been implicated in the ability of leptin to improve peripheral insulin sensitivity by its actions in the hypothalamus. The association of leptin with HOMA-IR was marginal; some other studies also showed an association [36], but other reports in obese adults and adolescents did not find an association [37].

Another important factor related to IR is triglyceride levels, which were associated with HOMA-IR but not IGFBP-1. However, in the analysis of IGFBP-1, removal of HOMA-IR from the model permitted the association with triglycerides to emerge. This means that hypertriglyceridemia has a stronger association with whole-body IR than hepatic IR. One factor contributing to hypertriglyceridemia is the inability of insulin to inhibit the release of VLDL from the liver [38]. The contribution to hypertriglyceridemia by *de novo* fatty acid synthesis in other tissues such as fat, muscle and intestines requires peripheral IR [39]. Genetic factors also affect hypertriglyceridemia. The PNPLA3 I148M variant may determine triglyceride profiles independent of obesity, supporting the idea that the I148M variant hampers intrahepatocellular lipolysis rather than stimulates triglyceride synthesis [40].

Another explanation for the lack of association of serum triglyceride levels with hepatic IR is that Notch 1 activity increases the intracellular abundance of triglycerides without an effect on serum lipids or VLDL secretion [41].

We found a strong association of whole-body IR with the mother's BMI, as previously reported, and interpreted this association to mean that inheritance, as well as shared family environment and lifestyle, are important determinants of child adiposity [42].

In regard to gender, girls had higher HOMA-IR values, as reported in other studies [43]. The possible influence of pubertal activation could not be analyzed because only seven girls showed stage 2 thelarche.

We found a negative association of IGFBP-1 with IGF-I, probably as a result of the dynamics of hormone receptor interaction, but this process may also result from the proteolysis of IGFBP-1 [44].

In the univariate analysis, the group with hepatic IR reported fewer hours of sleep. A previous report showed an association between short sleep duration and increased BMI and adverse metabolic outcomes in school children [45] but not with actual obesity at adolescence [46]. Rehman et al. [47] found higher IGFBP-1 levels with increased sleep, irrespective of sleep timing, although sleeping during the day resulted in higher levels of IGFBP-1. Appropriate sleep preservation may be an important strategy to promote healthy metabolic conditions.

As expected, acanthosis nigricans was associated with HOMA-IR. This finding is in accordance with previous studies [48]. This alteration may result from the interaction of increased insulin levels with IGF-1, triggering the proliferation of keratinocytes and fibroblasts [49].

NAFLD is associated with obesity. Overall, 42.6% of the children had ultrasonographic images showing a fatty liver. IR is considered an essential pathophysiological factor in the development of NAFLD [50]. Unexpectedly, in our study HOMA-IR was not associated with fatty liver. In agreement with this, a recent study reported that HOMA-IR was not independently associated with fatty liver in obese adolescents [51]. Furthermore, it has been suggested that steatosis is dissociated from insulin resistance in the I148M variant of PNPLA3 [52]. Therefore, we suggest that the direct association of fatty liver with HOMA-IR needs further investigation.

Previous studies showed that children with NAFLD have elevated leptin levels [53]. In our work, the analysis of variance showed higher leptin levels in children with fatty liver. We found that age associated with fatty liver. Kitajama et al. reported age associated with the severity of NAFLD in adults [54]. Waist girth was also associated with fatty liver as previously reported [55].

In this work, we also tested the interaction of three indices of adiposity with insulin resistance and associated factors. There is no agreement on the most appropriate estimator of obesity for metabolic evaluation in children. In adults, VAI is reported to be a good estimator of IR [56]. However, in children VAI seems to be inferior to BMI in terms of association with IR [57]. Some reports indicate that waist-to-height ratio is a sensitive marker of IR in children [58], but others indicate that this index is not superior to BMI in predicting metabolic or cardiovascular risk [59]. Our results are in agreement with the proposal that BMI is the best measure of adiposity associated with HOMA-IR in school children.

In the regression analysis, the waist-to-height ratio was the only index associated with IGFBP-1 levels. Previously, waist girdle was reported to be associated with low IGFBP-1 [60].

In summary, we found that 73.1% of obese children had whole-body and hepatic IR, indicating a strong interaction of these two physiopathological processes. These children were older than those without any type of IR. In regard to indices of adiposity, we found that BMI best predicts whole-body IR. In contrast, waist-to-height ratio seems to be the best index to predict hepatic IR, indicating that central obesity is critical for this condition. Leptin was the only factor associated with both whole-body and hepatic IR, but the significance of the association with hepatic IR was stronger. Triglyceride levels were related independently to whole-body IR. The mother's BMI was a predictor of children's HOMA-IR, showing the influence of genetic or early environment influences. IGF-I levels was another determinant of IGFBP-1. We did not find an independent association of fatty liver with IR in children.

## Supporting Information

Table S1 **Contains information on the comparison of groups with high or low HOMA-IR and IGFBP-1.**
(XLSX)

Table S2 **Contains information on the comparison of NAFLD groups.**
(XLSX)

Data S1 **Contains raw data.**
(XLSX)
